# Reducing Unnecessary Suicide Precautions in Non-Psychiatric Settings: A Quality Improvement Project in a Tertiary Healthcare Institution in Singapore

**DOI:** 10.1192/j.eurpsy.2026.10967

**Published:** 2026-07-31

**Authors:** S. H. Sekar, R. Y. Chee, C. Chang

**Affiliations:** Psychological Medicine, National University Hospital, Singapore, Singapore

## Abstract

**Introduction:**

Suicide precaution (SP) status is a critical safety measure but is often continued unnecessarily in non-psychiatric settings, even after psychiatric review. This practice places strain on nursing manpower, increases healthcare costs, and negatively affects patient dignity, autonomy, and therapeutic engagement. Literature on the effectiveness of constant observation and SP is limited, with studies highlighting both protective and adverse consequences. Addressing this issue is essential to balance safety with patient-centred care.

**Objectives:**

To reduce the proportion of patients in non-psychiatric settings at a tertiary healthcare institution in Singapore who remain on SP for more than 24 hours after psychiatric consult-liaison (CL) team review (without recommendation to continue) from 80% to 40% within 6 months.

**Methods:**

This quality improvement project will include adult inpatients (≥18 years) outside of psychiatric wards placed on SP and referred to the psychiatric CL team, excluding patients with <24 hours length of stay or those transferred to specialist psychiatric ward. Baseline data from May–November 2024 showed that 40 of 50 eligible cases (80%) had unnecessary SP continuation. Data will be collected via electronic reporting tools within the medical records system. Proposed interventions include:

- Education and communication: reinforcing appropriate use of SP through multidisciplinary teaching and clearer documentation in CL team recommendations.
- Safe Environmental Modifications (SEM): psychiatric CL team recommending bedside/cubicle modifications for lower-risk patients as an alternative to full SP.
- Regular review by psychiatric resource nurses within 48 hours to ensure timely discontinuation when no longer required.

**Results:**

Baseline findings demonstrated unnecessary SP continuation in 80% of reviewed cases. Post-intervention outcomes, monitored over 6 months, aim to reduce this figure to 40%. Secondary outcomes include improved patient satisfaction and reduced nursing burden.

**Conclusions:**

Unnecessary continuation of SP in non-psychiatric settings is common, resource-intensive, and detrimental to patient experience. This quality improvement initiative introduces structured interventions to reduce inappropriate SP orders, aligning safety practices with evidence-based, patient-centred care. Findings may inform institutional policies and contribute to broader discussions on optimizing suicide prevention strategies in general hospital settings.

**Disclosure of Interest:**

None Declared

